# First whole-genome chikungunya virus sequence detected in mosquitoes during the 2025 Foshan outbreak: evidence of field vector infection and transmission potential in China

**DOI:** 10.1186/s40249-025-01383-9

**Published:** 2025-10-23

**Authors:** Xinyu Zhou, Xiaoxue Xie, Wenhao Wang, Heting Gao, Kai Wang, Xiaohui Liu, Xiaoli Chen, Yuting Jiang, Haotian Yu, Dan Xing, Teng Zhao, Chunxiao Li

**Affiliations:** State Key Laboratory of Pathogen and Biosecurity, Beijing, China

**Keywords:** Chikungunya virus, *Aedes albopictus*, Whole-genome sequencing, Outbreak, Genomics, Foshan, China

## Abstract

**Background:**

Since July 2025, an outbreak of mosquito-borne chikungunya fever has occurred in Foshan City, Guangdong Province, China. This was the second large-scale local outbreak in China after the one that occurred in Dongguan City, Guangdong Province, in 2010. As of 23 August, more than 10,000 human cases had been reported. This study aims to investigate mosquito infection and viral genomic characteristics during the Foshan outbreak.

**Methods:**

Adult *Aedes albopictus* were collected using BioGents Sentinel trap in three hotspot towns (Beijiao, Chencun and Lecong). Mosquitoes were morphologically identified and pooled by species, sex and environment type. Each pool was homogenized, and the homogenate was clarified by centrifugation; and the supernatant was used for viral RNA isolation. The isolated RNA was screened using CHIKV RT-qPCR. Positive pools underwent Sanger sequencing and whole-genome sequencing. The CHIKV lineage and mutational profiles were inferred using maximum likelihood phylogenetic analysis and comparison with human- and mosquito-derived genomes.

**Results:**

Over 11 days of trapping, 2803 mosquitoes were captured. 1569 (55.97%) female *Ae. albopictus* were divided into 77 pools and 9.09% (7/77) of these pools were CHIKV-positive. The minimum infection rate (MIR, per 1000 females) for local *Ae. albopictus* was 4.46, while the MIR for residences in Lecong Town was the highest at 9.17 per 1000 females. The MIR for parklands was slightly higher than for residences (4.60 vs. 4.30 per 1000 females). Five complete *Ae. albopictus*-derived CHIKV genome clustered within the East/Central/South African-Indian Ocean lineage genotype, and harbored novel E1 and E2 mutations consistent with those detected in the 2025 Reunion Island human strain. Amino-acid mutations E1-A226V/E2-L210Q were detected, enhancing adaptability to *Ae. albopictus* and increasing the transmission capacity.

**Conclusions:**

This study represents the first mosquito-derived CHIKV whole-genome sequence obtained from the 2025 Foshan outbreak. *Ae. albopictus* was confirmed as the primary vector, and the presence of adaptive mutations indicated an enhanced transmission potential. Despite the outbreak emerging earlier in the season and affecting a dense urban population, it was effectively controlled through timely and intensive vector interventions. These findings highlighted the critical role of mosquito surveillance in early outbreak preparedness and effective vector management.

**Graphical Abstract:**

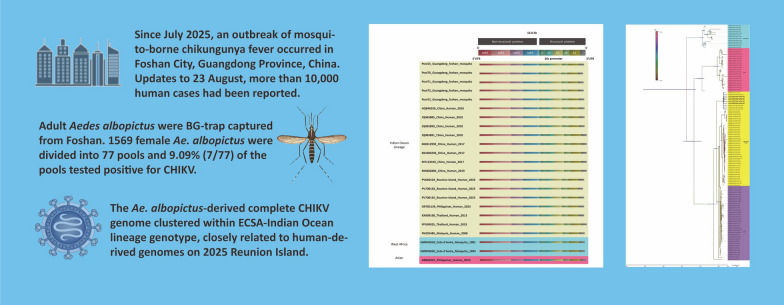

**Supplementary Information:**

The online version contains supplementary material available at 10.1186/s40249-025-01383-9.

## Background

The chikungunya virus (CHIKV) is a mosquito-borne single-stranded RNA virus (belonging to the *Togaviridae* family, *Alphavirus* genus) that causes an acute febrile illness accompanied by severe and debilitating arthralgia [1]. The primary vectors for CHIKV are *Aedes aegypti* and *Ae. albopictus* [2]. Given the widespread distribution of *Aedes* mosquitoes across tropical and subtropical regions, CHIKV has demonstrated significant global cross-regional transmission potential [3]. CHIKV is currently classified into four major genotypes: West African (WA), East/Central/South African (ECSA), Asian, and the Indian Ocean Lineage (IOL, a branch of the ECSA genotype). Since its first detection in Tanzania in 1952, CHIKV has been sporadically confined to Asia and Africa. However, since the early twenty-first century, it has re-emerged in over 100 countries across Asia, Africa and America. For example, the introduction of IOL genotype had led to explosive epidemics in India and Southeast Asia, posing a serious threat to public health [4–6].

In 2010, Dongguan City in Guangdong witnessed China’s first large-scale local CHIKV outbreak, where existed abundant *Ae. albopictus* populations. The Dongguan CHIKV strain belonged to the ECSA-IOL genotype with the E1-A226V mutation, which significantly enhances its adaptability to the local primary vector *Ae. albopictus* [7, 8]. Subsequently, imported cases and sporadic local transmission were reported in Yunnan, Zhejiang, and other regions of China [9, 10]. The emerging outbreak in Foshan City, Guangdong Province, which began in July 2025, has infected above 10 thousand people by August 23 [11]. The subtropical climate and rapid urbanization in China, combined with high *Aedes* mosquito activity and population density, have amplified the CHIKV transmission risk in the Pearl River Delta region (Guangdong Province).

Viral genomes were instrumental in the identification of the epidemics origin and the reconstruction of transmission chains [12]. However, most CHIKV viral sequences from the Foshan 2025 outbreak were derived from patients, and no field mosquito-derived CHIKV genome has been reported, which hindered comprehensive understanding of the transmission ecology and the establishment of phylogenetic linkage between human cases and local vector. Several mosquito-derived CHIKV genomes have been documented from epidemics in Asia, Europe and Latin America countries [13–16]. Mosquito-derived CHIKV genomes not only confirmed vector infection but also revealed potential adaptive mutations. The E1-A226V mutation, first identified in the IOL strains during the 2005–2006 Indian Ocean outbreak, dramatically increased CHIKV infectivity and dissemination in *Ae. albopictus*. In 2009, a second-step mutation (E2-L210Q) emerged in India, further enhancing midgut infection in *Ae. albopictus* without affecting fitness in *Ae. aegypti*. These sequential adaptations have enabled CHIKV to exploit *Ae. albopictus* as a more efficient urban vector across a wider geographic and climatic range, where *Ae. albopictus* predominates, such as in urban China [17, 18].

This study was conducted during the ongoing CHIKV outbreak Foshan, China, in August 2025. In this study, the CHIKV positivity rate in *Aedes* mosquitoes was detected, and the first whole genome of mosquito-derived CHIKV strains was obtained. By analyzing mosquito infection rates, genotypes, and key mutation profiles, this study provided critical evidence for CHIKV vector attribution and an evaluation of the mosquito control efficacy during the outbreak, enabling timely responses and guidance for mosquito-borne disease control measures.

## Methods

### Sampling sites

This study was conducted in the field area of Beijiao, Chencun and Lecong Towns in Shunde District, Foshan City, Guangdong Province, China, which were hotspot regions (over 90% of patient cases were reported at the initial phase) for the CHIKV pandemic (Supplementary Material 1). The study area lies approximately 30–40 km from Guangzhou City (international city with 19 million population) and has a subtropical humid climate with an annual average temperature of 22.2 °C (range 10 °C–33 °C). The region receives approximately 1677.3 mm of precipitation annually, with June having the highest precipitation at 273.7 mm and an annual average relative humidity of 79%, which is highly suitable for the survival of the primary vector *Aedes* mosquitoes.

### Mosquito collection and processing

The mosquitoes were collected outdoors with BioGents Sentinel trap (BG-trap) (Biogents, Regensburg, Germany) from 9:00 am to 8:00 pm from July 31st to Aug 10th 2025. Adult mosquitoes were frozen at − 20 °C for 30 min and placed on ice for morphological identification, then immediately transferred in carbon dioxide ice. Taxonomically diagnostic characteristics included cephalic structures (antennal morphology and maxillary palp features), thoracic scutal patterns, abdominal markings, wing venation architecture, and leg banding coloration, following established entomological keys. For example, *Ae. albopictus* has a single, longitudinal, silvery dorsal stripe, while *Ae. aegypti* has a lyre-shaped (paired) dorsal pattern on its scutum.

We restricted analyses to female *Aedes* mosquitoes, as only females blood-feed and contribute to arbovirus transmission. the *Aedes* mosquitoes were quantified and grouped in pools, according to species, sex, location. Each pool comprised approximately 25 individuals. Each pool was homogenized by motor driven tissue grinder with 1 ml of Media Dulbecco’s Modified Eagle Medium (DMEM) (Thermo Fisher Scientific, Waltham, USA) supplemented with 2% fetal bovine serum (FBS) (Thermo Fisher Scientific, Waltham, USA). Following homogenization, the samples were centrifuged at 8000 × *g* for 10 min at 4 °C. The supernatant was subsequently collected and stored at −80 °C until further processing.

### Viral RNA extraction and CHIKV molecular detection with RT-qPCR

The prepared homogenate of mosquitoes was clarified by centrifugation and the supernatant was used for viral RNA isolation. RNA was extracted from pools of clarified mosquito homogenates using viral RNA isolation kit (QIAGEN, Duesseldorf, Germany), according to manufacturer’s instructions. The RNA was eluted from the QIAspin (QIAGEN, Duesseldorf, Germany) columns in a final volume of 80 μl of ddH_2_O and was kept at − 80 °C until processing.

The reverse transcription quantitative polymerase chain reaction (RT-qPCR) assay was performed using the CHIKV Detection Kit (Slin Medical Technology, Guangzhou, China), which contains specific primers and a probe targeting the CHIKV E1 gene. Each 25 μl reaction contained 20 μl of reaction mix and 5 μl of extracted RNA. Amplification was carried out on an Applied Biosystems^®^ QuantStudio^™^ 7 Flex Real-Time PCR System (ThermoFisher Scientific, Waltham, MA, USA) under the following conditions: reverse transcription at 50 °C for 2 min, initial denaturation at 95 °C for 1 min, followed by 40 cycles of denaturation at 95 °C for 2 s and annealing/extension at 55 °C for 19 s. A plasmid containing a fragment of the CHIKV E1 gene was used as a positive control, and nuclease-free water was included as a negative control. All samples were run in duplicate, and those with an average cycle threshold (Ct) value below 37 were considered as positive.

### Sanger sequencing of mosquito-derived CHIKV genomes

Full-length CHIKV genomes were amplified using overlapping RT-PCR with primer sets designed based on S27 strain reference sequences from GenBank (accession GCA_000854045.1). Full viral genome recovery was achieved via synthesis of complementary DNA (cDNA) directly from single-stranded RNA (ssRNA). Briefly, cDNA was synthesized using PrimeScript^™^ RT Master Mix (TAKARA, Tokyo, Japan). The CHIKV genome was sequenced through overlapping PCR amplicons spanning 12 genomic segments. All primers used for amplification (Table 1) were commercially synthesized by Sangon Biotech (Sangon, Shanghai, China), with subsequent PCR product sequencing performed by the same vendor. Raw chromatograms were assembled into contiguous sequences using SeqMan Pro Lasergene v7.1 (DNASTAR, Madison, USA) and manually curated to generate the complete genome sequences.

**Table 1:** Primers for sanger sequencing of mosquito-derived chikungunya virus genomes

No	Region (bp)	Forward primer (5′ → 3′)	Reverse primer (5′→ 3′)
1	1–943	ATGGCTGCGTGAGACA	TCTCTTAACGACGTAGCCTT
2	713–2005	TGTTCAACAGACCTGACGGA	CATCGCAATATGGTGTAGCTT
3	1917–3160	CTGAAGACTTCCAGAGCCTA	TTTTATCCCCGCTGTTTCGAG
4	2680–3921	GAATGAGTACAACAAGCCGAT	TGATCTAAACTTGCGTCCCA
5	3762–5126	ATTATCAACAGTGCGTAGACCA	CGTCAACGCTTAGATCGAAT
6	4869–6343	GTTACGCCATGACACCAGA	CTCCACGTTGAATACTGCT
7	5592–6905	ATGATTTGACAGATAGCGACT	AGCTGGAAATCTCTCCGAA
8	6795–8039	AGAGCCAAGATGATTCACTTGC	GATGACCGCTTAAAGGCCAA
9	7994–9145	CCATCGATAACGCGGACCTG	TACACTTATACCGCACCGTCT
10	8634–10,046	CGTAGCACTAGAACGCATC	AGAGTCTTATACGGTACTCCC
11	9803–11,290	CTAAAGCGGCCACATACCAA	ATAGCACCACGATTAGAATCAG
12	10,778–12,032	CAACAAACCCGGTAAGAGC	GAAATATTAAAAACAAAATAACATCTCCTACGTC

### Next-generation sequencing of mosquito-derived CHIKV genome

Due to the low viral RNA concentration, some PCR-positive pools were subjected to high-throughput sequencing. cDNA libraries were prepared using the NEBNext Ultra RNA Library Prep Kit (New England Biolabs, Ipswich, USA) for Illumina. The libraries were quality checked and sequenced on an Illumina Nova Seq 6000 platform (Illumina, San Diego, USA) with 150-bp paired-end reads.

The analysis pipeline for high-throughput sequencing data was as follows: First, Trimmomatic 0.39 (Max-Planck-Gesellschaft, Berlin, Germany) [19] and Fastp 0.23.4 (HaploX, Shenzhen, China) [20] were used to remove sequencing adapters and low-quality sequences, completing the sequencing data quality control. Next, Bowtie2 2.5.4 (University of Maryland, Maryland, USA) [21] was employed in conjunction with the *Ae. albopictus* reference genome (Accession Number: GCF035046485.1) [22] to perform host sequence depletion on the quality-controlled sequencing results. Subsequently, the host-depleted sequences were aligned against the chikungunya virus genome sequence (Accession Number: NC_004162.2) [23] using ncbi-blast + 2.16.0 (National Center for Biotechnology Information, Bethesda, USA) [24], and chikungunya virus-specific sequences were isolated from the alignment output. These isolated viral sequences were then assembled with MEGAHIT 1.2.9 (University of Hong Kong, Hong Kong, China) [25]. For the assembled contigs, BWA 0.7.17-r1188 (Wellcome Trust Sanger Institute, Cambridge, UK) [26] was used to evaluate the sequencing coverage depth, and ncbi-blast + 2.16.0 (National Center for Biotechnology Information, Bethesda, USA) for sequence homology alignment. Finally, the final high-quality assembly results were screened and obtained through these validation steps.

### Phylogenetic analysis

Besides the five mosquito-derived CHIKV strains detected in Foshan, an additional 125 complete genomes of chikungunya virus were collected and curated, with isolation sources including mosquitoes and humans (Supplementary material 2). It should be noted that, as the sudden outbreak, no sequences from local patients in Foshan 2025 had been found in the database. Following codon-based multiple sequence alignment using MUSCLE 3.8.1551 (Robert Edgar, Sonoma, USA) [27], the Bayesian Evolutionary Analysis Utility (BEAUti) 10.5.0 (University of California, Los Angeles, USA) [28] was used for evolutionary model selection and XML (Extensible Markup Language) file construction.

Bayesian Evolutionary Analysis Sampling Trees (BEAST) 10.5.0 (University of California, Los Angeles, USA) [29] was employed to estimate evolutionary rates, divergence times, population sizes, and tree topologies. Tracer 1.7.2 (The University of Edinburgh, Edinburgh, UK) [30] was utilized to assess the convergence of phylogenetic tree topology parameters.

TreeAnnotator 10.5.0 (University of California, Los Angeles, USA) [31] was used to summarize the posterior estimates and highest posterior density (HPD) limits of node heights, as well as evolutionary rates for analyses employing a relaxed molecular clock model. Finally, FigTree 1.4.4 (The University of Edinburgh, Edinburgh, UK) was applied to visualize and refine the phylogenetic results for presentation purposes.

### Amino acid mutation analysis

For sequence variant detection, the complete genomes of each Foshan mosquito-derived CHIKV strain were aligned to the typical genotypes from human and mosquito reference strains using ClustalW 1.8.1 (Institut de Génétique et de Biologie Moléculaire et Cellulaire, Illkirch Cedex, France) [32], and the bases of each Foshan mosquito-derived CHIKV strain virus genome that did not align were extracted as single-nucleotide variants (SNVs) using custom-written Python scripts. These SNVs were annotated by ANNOVAR software 2025Mar21 (Columbia University, New York, USA), and SNVs in the coding region were divided into synonymous SNVs and nonsynonymous SNVs.

### Comparative genomic analysis

BLASTn (National Center for Biotechnology Information, Bethesda, USA) was used to predict the sequences and genomic positions of structural and non-structural proteins of chikungunya virus, and the genome structure was plotted using R package circlize 0.4.16 (Heidelberg University, Baden-Württemberg, Germany) [33].

### Minimum infection rate

For infection rate estimation, we restricted analyses to female *Aedes* mosquitoes, as only females blood-feed and contribute to arbovirus transmission. Pools were formed with around 25 individuals, and infection rates were expressed as minimum infection rate (MIR, per 1000 females). The MIR was determined as the ratio between the number of positive pools of mosquitoes detected and the total number of mosquitoes tested, multiplied by 1000. This approach has been widely adopted in arbovirus entomological surveillance to avoid dilution effects from non-vector males, which may bias estimates downward.

## Results

### Mosquito collection and identification

Sampling sites were selected from three hotspot towns (Beijiao, Chencun and Lecong) in Foshan City, Guangdong Province, China, including 56 parklands and 34 residential areas. A total of 2803 mosquitoes (1031 in Beijiao, 500 in Chencun and 1272 in Lecong) were trapped in the study. The majority were *Ae. albopictus* (2627, 93.72%), with the others being *Culex* spp., with no *Ae. aegypti.* There were 1087 female *Ae. albopictus* trapped from parklands and 465 from residences. (Supplementary material 1).

### CHIKV infection rate in mosquito

We focused on female *Aedes* mosquitoes, as only females blood-feed and contribute to arbovirus transmission. There were 1569 (59.73%) female *Ae. albopictus* from 90 sites in 3 towns, which were divided into 77 pools by location and environmental type. There were 9.09% (7/77) of the pools tested positive for CHIKV (Table 2).

**Table 2:** Chikungunya virus positive pool information for female *Aedes albopictus* mosquitoes

Pool code	Code of sampling site	Number of female *Ae. albopictus*	Town	Environmental type	Sampling date	RT-PCR Ct value
P12	S14	20	Lecong	Residences	2025-08-01	21.63
P15	S15, S16	23	Lecong	Residences	2025-08-01	20.57
P22	S25	20	Lecong	Parklands	2025-08-02	35.14
P23	S27	15	Lecong	Parklands	2025-08-02	35.68
P70	S83	20	Beijiao	Parklands	2025-08-10	21.61
P71	S83	20	Beijiao	Parklands	2025-08-10	20.39
P72	S85, S86	24	Beijiao	Parklands	2025-08-10	21.40

*RT-PCR* reverse transcription-PCR

The total minimum infection rate (MIR, per 1000 females) was 4.46, while the MIR for residences in Lecong Town was the highest at 9.17. The MIR for parklands was slightly higher than for residences (4.60 vs. 4.30, Table 3).

**Table 3:** Minimum infection rate (MIR) of chikungunya virus in mosquito from different towns and regional types

Town	Environmental type	Total of female *Ae. alcbopictus*	Total of the pools	No. of pools positive by RT-PCR (%)	MIR per 1000 female
Beijiao	Parklands	437	21	3 (14.28)	6.86
	Residences	162	8	0	0
Chencun	Parklands	217	11	0	0
	Residences	85	4	0	0
Lecong	Parklands	450	22	2 (9.09)	4.44
	Residences	218	11	2 (18.18)	**9.17**
Total	Parklands	1087	54	5 (9.26)	4.60
	Residences	465	23	2 (8.70)	4.30
	Total	1569	77	7 (9.09)	**4.46**

*RT-PCR* reverse transcription-PCR

### Phylogenetic analysis

**Fig. 1:**
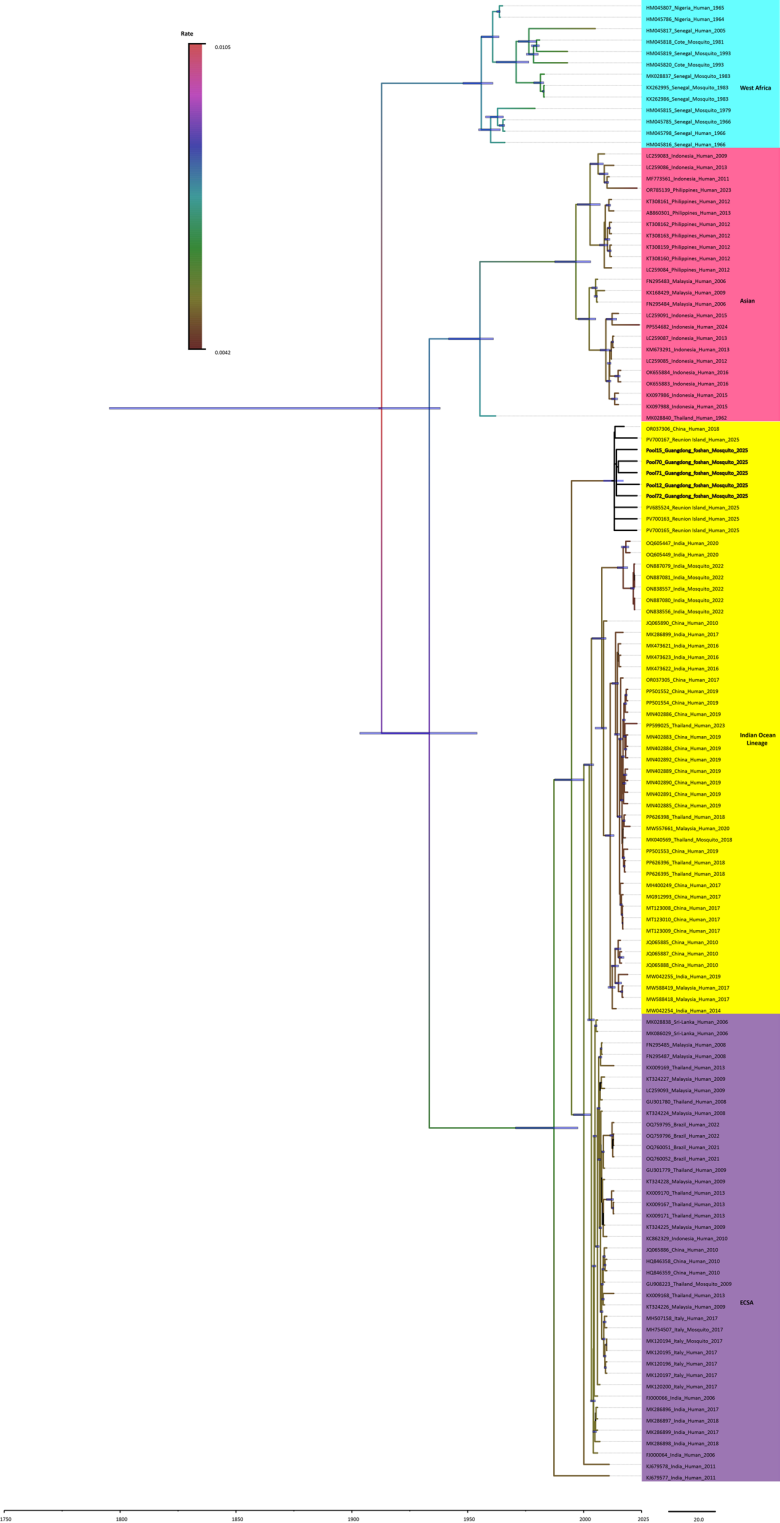
Maximum clade credibility (MCC) tree of 130 CHIKV strains. The four major lineages were highlighted with different branch colors, where the color of each branch line represented the evolutionary rate of the viruses in that branch. The estimated 95% highest posterior density (HPD) values for most recent common ancestors were labeled beside the node and were also indicated by the thick blue horizontal node bars. The numbers adjacent to nodes indicated Bayesian posterior probability values. Strains were labeled as follows: strain_name_location_host_date (year) of collection. The 5 Foshan mosquito-derived CHIKV strains in this study were in bold. *CHIKV* chikungunya virus, *HPD* highest posterior density

There were five whole genome sequences finally acquired (GenBank access number PX 399440 to PX 399444), with the PCR-positive pools excluding 2 low-viral-load pools (CT value beyond 35). The initial Maximum clade credibility tree was constructed using the dataset containing 135 sequences from the four distinct genotypes and the Foshan mosquito-derived sequences in the study. Phylogeny based on the complete genome analysis characterized the five Foshan mosquito-derived CHIKV strains (12/15/70/71/72_Guangdong_foshan_Mosquito_2025), as belonging to the ECSA-IOL genotype, with a high similarity to the Réunion Island human case in 2025 (99.93%) and local human case in 2018 (97.01–97.04%). It comprised a distinct sub-branch with other mosquito derived samples previously detected in Guangdong, Zhejiang and Yunnan (Fig. 1). This showed a potential genetic origin of the virus strains in this outbreak.

Phylogenetic results indicated that all currently circulating CHIKV strains share a common ancestor that existed within the past 300 years, with the 95% highest posterior density (HPD) interval for their most recent common ancestor (MRCA) estimated to be 87–230 years ago. For CHIKV strains endemic to the Asian region, the 95% HPD interval for their MRCA ranges from 71 to 122 years ago. The divergence between the Asian and ECSA genotypes occurred within the past 100 years. Furthermore, phylogenetic results revealed a distinct spatiotemporal pattern in the Southeast Asian lineage of the Asian genotype: it spread from Thailand to Indonesia, subsequently to the Philippines, and most recently to Malaysia. The most recent common ancestor (MRCA) of the Indian Ocean lineage can be traced back to approximately 2002 (95% HPD: 2001–2003).

### Amino acid mutation analysis

The mutation analysis, compared to human- and mosquito-derived strains, is shown in Table 4 for four genotypes of CHIKV structural proteins. All consensus genomes assigned to the five Foshan 2025 mosquito-derived CHIKV strains (Pools 12, 15, 70, 71 and 72) displayed an identical E1/E2 amino-acid signature, indicating a single predominant circulating variant in local vectors.

**Table 4:** Mutations in Foshan mosquito-derived CHIKV strains, compared with typical sequences from human and mosquito in the four genotypes

Virus strain	Origin	Location	Year	E1 mutation										E2 mutation														Genotype
				9	37	211	226	250	284	317	324	348	399	74	85	118	149	205	210	222	227	246	264	282	312	375	386	
Pool15	Mosquito	Foshan, China	2025	**S**	**I**	**K**	**V**	**P**	**D**	**V**	**R**	**E**	**I**	**T**	**A**	**G**	**R**	**G**	**Q**	**I**	**V**	**D**	**V**	**K**	**T**	**S**	**V**	ECSA-IOL
Pool12	Mosquito	Foshan, China	2025	**S**	**I**	**K**	**V**	**P**	**D**	**V**	**R**	**E**	**I**	**T**	**A**	**G**	**R**	**G**	**Q**	**I**	**V**	**D**	**V**	**K**	**T**	**S**	**V**	ECSA-IOL
Pool70	Mosquito	Foshan, China	2025	**S**	**I**	**K**	**V**	**P**	**D**	**V**	**R**	**E**	**I**	**T**	**A**	**G**	**R**	**G**	**Q**	**I**	**V**	**D**	**V**	**K**	**T**	**S**	**V**	ECSA-IOL
Pool71	Mosquito	Foshan, China	2025	**S**	**I**	**K**	**V**	**P**	**D**	**V**	**R**	**E**	**I**	**T**	**A**	**G**	**R**	**G**	**Q**	**I**	**V**	**D**	**V**	**K**	**T**	**S**	**V**	ECSA-IOL
Pool72	Mosquito	Foshan, China	2025	**S**	**I**	**K**	**V**	**P**	**D**	**V**	**R**	**E**	**I**	**T**	**A**	**G**	**R**	**G**	**Q**	**I**	**V**	**D**	**V**	**K**	**T**	**S**	**V**	ECSA-IOL
PV685524	Human	Reunion Island	2025	**S**	**I**	**K**	**V**	**P**	**D**	**V**	**R**	**E**	**I**	**T**	**A**	**G**	**R**	**G**	**Q**	**I**	**V**	**D**	**V**	**K**	**T**	**S**	**V**	ECSA-IOL
PV700165	Human	Reunion Island	2025	**S**	**I**	**K**	**V**	**P**	**D**	**V**	**R**	**E**	**I**	**T**	**A**	**G**	**R**	**G**	**Q**	**I**	**V**	**D**	**V**	**K**	**T**	**S**	**V**	ECSA-IOL
MG912993	Human	Zhejiang, China	2017	N	T	E	A	S	E	**V**	K	G	V	M	V	S	K	S	L	V	A	A	A	Q	M	T	A	ECSA-IOL
JQ065885	Human	Guangdong, China	2010	N	T	**K**	**V**	**P**	E	I	K	G	V	M	V	S	K	**G**	L	V	A	A	**V**	Q	M	T	A	ECSA-IOL
MN402884	Human	Yunnan, China	2019	N	T	E	A	S	E	**V**	K	G	V	M	V	S	K	S	L	V	A	A	A	Q	M	T	A	ECSA-IOL
JQ065890	Human	Guangdong, China	2010	N	T	E	A	S	E	I	K	G	V	M	V	S	K	**G**	L	V	A	A	A	Q	M	T	A	ECSA-IOL
HQ846356	Human	Guangdong, China	2010	N	T	**K**	**V**	**P**	E	I	K	G	V	M	V	S	K	**G**	L	V	A	A	**V**	Q	M	T	A	ECSA-IOL
PP599025	Human	Thailand	2023	N	T	E	A	S	E	**V**	K	G	V	M	V	S	K	S	L	V	A	A	A	Q	M	T	A	ECSA-IOL
KX009168	Human	Thailand	2013	N	T	**K**	**V**	S	E	I	K	G	V	M	V	S	K	**G**	L	V	A	A	**V**	Q	M	T	A	ECSA-IOL
ON887079	Mosquito	India	2022	N	T	E	A	S	E	I	K	G	V	M	V	S	K	**G**	L	V	A	A	A	Q	M	T	A	ECSA-IOL
FN295485	Human	Malaysia	2008	N	T	**K**	**V**	S	E	I	K	G	V	M	V	S	K	**G**	L	V	A	A	**V**	Q	M	T	A	ECSA-IOL
AB860301	Human	Philippines	2013	N	T	E	A	S	**D**	I	K	G	V	M	V	**G**	**R**	D	L	V	A	A	**V**	Q	**T**	**S**	**V**	Asian-Pacific
HM045820	Mosquito	Cote d’Ivoire	1993	N	T	**K**	A	S	**D**	I	K	G	V	**T**	V	S	K	**G**	L	**I**	A	A	**V**	Q	**T**	**S**	**V**	WestAfrican
MK286898	Human	India	2018	N	T	E	A	S	E	V	K	G	V	M	V	S	K	G	L	V	A	A	A	Q	M	T	A	ESCA

Virus strain column represented the five Foshan mosquito-derived CHIKV strains (Pools 12, 15, 70, 71 and 72) and accession number for other sequences used in the mutation alignment. The mutated sites were highlighted in bold. *aa* Amino acids, *A* Alanine, *D* Aspartic acid, *E* Glutamic acid, *G* Glycine, *I* Isoleucine, *K* Lysine, *L* Leucine, *N* Asparagine, *P* Proline, *Q* Glutamine, *R* Arginine, *S* Serine, *T* Threonine, *V* Valine

Notably, this constellation included the E1-A226V and E2-L210Q mutations, which had been identified as enhancing *Ae. albopictus* adaptation and increasing infectivity. The novel E1-N9S/T37I/K324R/G348E/V399I mutation together with the E2-V85A/A227V/Q282K mutation was observed, which was identical to those observed in two contemporaneous Réunion Island human isolates (PV685524 and PV700165) in 2025, supporting a shared variant profile across regions and facilitating tracing.

Contrasting with earlier Chinese reference strains (e.g. Dongguan 2010 and Zhejiang 2017), the Foshan variant consistently replaced ancestral residues at multiple E1 sites (N9S, T37I, K324R, G348E and V399I) and E2 sites (V85A, A227V and Q282K). These changes made the 2025 Foshan mosquito-derived CHIKV different from former domestic lineages within ECSA–IOL.

### Comparative genomic analysis

**Fig. 2:**
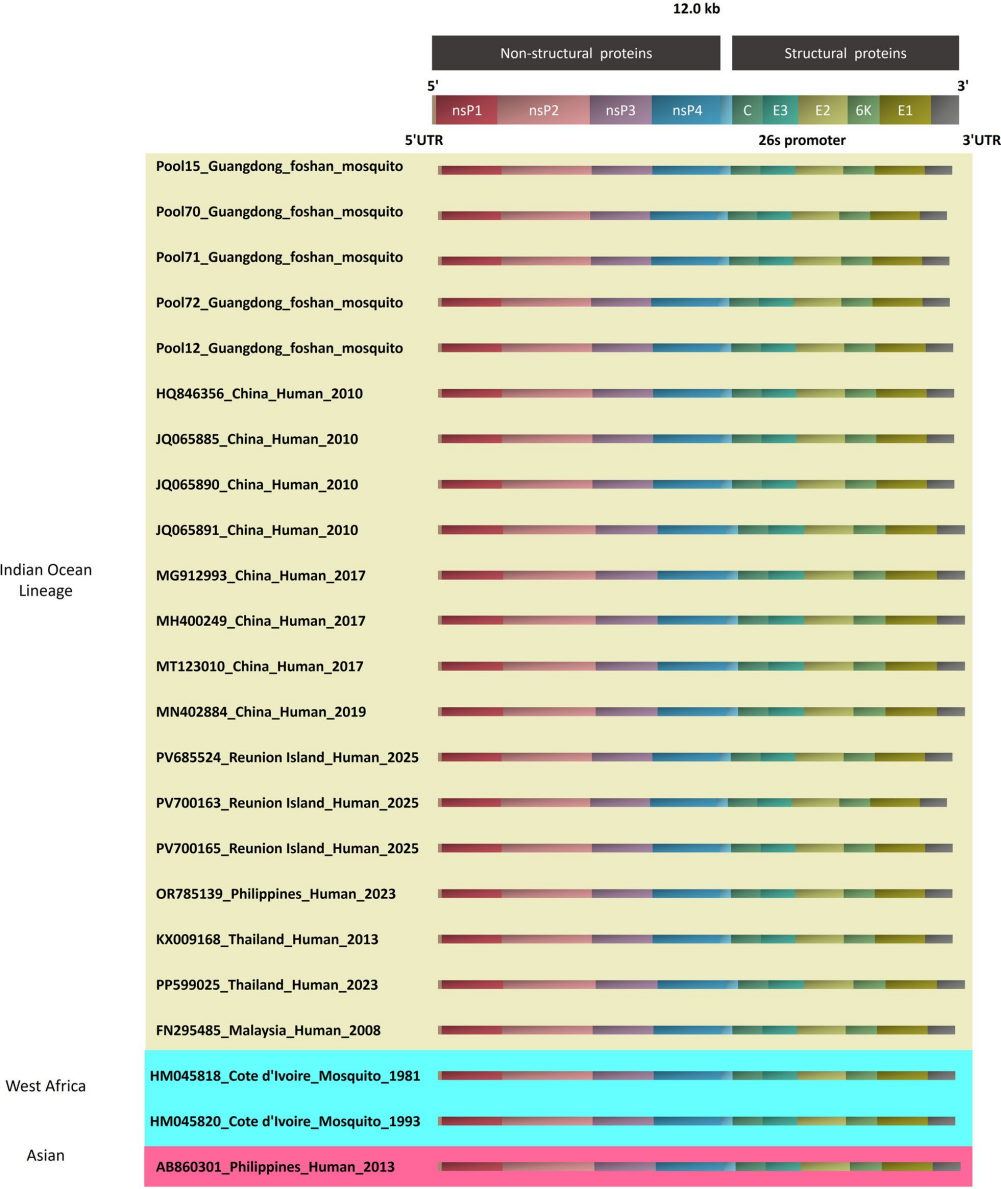
Comparative genomics analysis of 23 CHIKV strains. It showed the positions of gene-coding regions across different CHIKV lineages. Here, nsP represented for non-structural proteins, E for Envelope proteins, C for Capsid protein, and UTR for Untranslated Regions. Different background colors were used to distinguish between different CHIKV genotypes. *CHIKV* chikungunya virus, *UTR* untranslated region

The complete sequencing of the five Foshan isolates and other 18 sequnences from patients and mosquitos were compared with nonsynonymous SNV, for a better understanding of their genetic relationships. The different regions of the sequences exhibited > 90.4% nucleotide similarity to the corresponding regions of the prototypical isolate (Fig. 2).

## Discussion

This study reported the first mosquito-derived complete CHIKV genome from *Ae. albopictus* during the 2025 Foshan China outbreak, providing direct evidence of the vector mosquito *Ae. albopictus* ‘s role in the CHIKV transmission. The observed mosquito infection rate (MIR = 4.46), together with adaptive mutations such as E1-A226V and E2-L210Q, provided timely and effective support for public health response.

Globally, CHIKV detection rates in *Aedes* mosquitoes consistently increased during epidemic periods compared with inter-epidemic phases. For instance, a large-scale epidemic in India from 2006 to 2010 reported the MIR of 2–15 per 1000 *Ae. aegypti* and *Ae. albopictus*, while routine surveillance typically detected fewer than 1 per 1000 [34, 35]. Studies from Thailand, Indonesia, Singapore and other Asian countries similarly demonstrated significant increases in *Ae. albopictus* positivity rates during outbreaks, reaching over 8% in some settings, compared with sporadic detections in non-outbreak years [36–38]. The situation for *Ae. aegypti* was similar. Latin American surveys, such as in Colombia (2020–2021), revealed very low positivity in *Ae. aegypti* during non-outbreak periods (0.2%), whereas epidemic periods in Brazil (2017–2020) reached 2–8 per 1000 [39, 40]. Higher infection levels were reported in Africa, such as in Kenya, where a rate exceeded 10 per 1000 [41]. Collectively, these findings confirmed that vector positivity rates rose sharply during outbreaks, and the Foshan outbreak MIR fell within the range observed elsewhere, suggesting comparable transmission dynamics and the efficacy of existing control measures.

Phylogenetic analysis showed that the five Foshan mosquito-derived CHIKV genomes clustered with the Réunion Island human case in 2025, belonging to the same ECSA-IOL genotype as Foshan Local human-derived CHIKV [11]. In addition, the Foshan mosquito-derived CHIKV amino-acid mutation on E1/E2 regions was identical to those observed in Réunion Island human isolates in 2025, supporting a potential genetic origin. Importantly, mutations such as E1-A226V and E2-L210Q, both detected in this study, were recognized as markers of enhanced vector adaptation. E1-A226V was reported to increase infectivity in *Ae. albopictu*s approximately 100-fold by facilitating midgut invasion and shortening the extrinsic incubation period [42]. Similarly, the E2-L210Q allele was associated with enhanced viral replication efficiency within the vector [43]. These mutations, first highlighted during the 2005–2006 Indian Ocean epidemic, later emerged independently in multiple regions, including Thailand and Malaysia [38, 44], reflecting selective pressure for *Ae. albopictus* adaptation. Conversely, outbreaks in the Americas largely involved Asian genotype viruses lacking E1-A226V, demonstrating regional variation in adaptive signatures [45]. The Foshan mosquito-derived CHIKV strains identified in this study carried the E1-A226V and E2-L210Q mutations, which were of significant importance in explaining the CHIKV adaptive transmission in local primary vector *Ae. albopictus*. Other E2 substitutions such as R198Q, K233E, and K252Q had also been shown to provide incremental fitness benefits in *Ae. albopictus* within IOL strains [46, 47]. Additional amino-acid changes observed in Foshan strains had not previously been implicated in vector adaptation so far.

Despite the early implementation of large-scale vector control during the Foshan outbreak, CHIKV-positive mosquitoes were still detected, which was consistent with observations in Brazil, Thailand and other regions, where wild-caught mosquitoes remained virus-positive even under intensive interventions. This persistence likely reflected challenges such as complex mosquito habitats, insecticide resistance [48, 49]. Comparisons with the concurrent 2025 Reunion Island outbreak were instructive: although Reunion experienced lower seasonal mosquito abundance, nearly one-quarter of the population was infected, with > 23,000 estimated cases per week [50, 51]. In Foshan, despite higher population density and favorable conditions for mosquito activity, timely interventions limited the cases to around 10,000, underscoring the critical role of rapid, intensive vector management. Otherwise, the outbreak might have resulted in greater spread and loss.

Our findings emphasized that mosquito-based surveillance was indispensable for outbreak preparedness. Incorporating systematic human/mosquito monitoring and viral genome sequencing into public health responses provided an early warning system, informed the detection of adaptive mutations, and enabled real-time evaluation of intervention efficacy. MIR estimates, when combined with genomic data, could have guided adjustments to control intensity and complemented case-based surveillance.

Compared to previous chikungunya outbreaks over the past decade, the Foshan outbreak occurred earlier (early July), affected a larger urban area (with a population of over 9.5 million), and presented abundant breeding sites for the vector mosquito *Ae. albopictus*. However, the number of cases so far exceeded 10,000, and the number of daily new cases remained under effective control, which was closely associated with the strong vector control measures implemented by the Chinese government in the early stages of the outbreak. Moreover, this event once again underscored the necessity of early monitoring of vector mosquitoes and the importance of implementing highly effective vector intervention measures as soon as possible after an outbreak occurred.

Despite the study’s objective to furnish the current data to support prevention during the outbreak period, it was undeniable that certain limitations remained. First, the estimated positivity rate might be constrained by the scope of the sampling and pooling strategies. Second, comprehensive experiments to isolate viruses and evaluate vector competence were not conducted. Further validation of the role of mosquitoes in the transmission chain would be conducted through longitudinal monitoring and vector competence experiments.

## Conclusions

This study provided the first complete CHIKV genome sequence obtained from mosquitoes during an ongoing outbreak in China since July 2025, thereby contributing essential entomological evidence to complement human case investigations. Furthermore, the analysis of mosquito positive rates and amino-acid mutation spectra directly provided crucial support for the process of tracing and risk assessment. It emphasized the importance of coordinated mosquito surveillance, in order to support a timely and effective public health response.

## Supplementary Information

Supplementary material 1. Mosquito collection information in 90 sampling sites, Foshan, China, 2025.

Supplementary material 2. Additional 125 complete genomes of chikungunya virus information for phylogenetic analysis.

## Data Availability

Five mosquito-derived CHIKV whole genome sequence in Foshan, China can be acquired through GenBank access number PX399440 to PX399444. Data and materials will be made available on request.

## Abbreviations

CHIKV: Chikungunya virus
RNA: Ribonucleic acid
RT-qPCR: Reverse transcription quantitative polymerase chain reaction
MIR: Minimum infection rate
ECSA: East/Central/South African lineage
E1: Envelope protein 1
E2: Envelope protein 2
IOL: Indian Ocean lineage
DMEM: Dulbecco’s modified eagle medium
FBS: Fetal bovine serum
PCR: Polymerase chain reaction
DNA: Deoxyribonucleic acid
NGS: Next-generation sequencing
nt: Nucleotide
aa: Amino acid
MRCA: Most recent common ancestor
MCC: Maximum clade credibility
HPD: Highest posterior density
UTR: Untranslated region
