# Supplementary material for: First whole-genome chikungunya virus sequence detected in mosquitoes during the 2025 Foshan outbreak: evidence of field vector infection and transmission potential in China
Source: Infect Dis Poverty. 2025 Oct 23;14:108. doi: 10.1186/s40249-025-01383-9 (PMC12548186; doi:10.1186/s40249-025-01383-9)
Supplement: Supplementary file 1 — Supplementary material 1. Mosquito collection information in 90 sampling sites, Foshan, China, 2025. [file 40249_2025_1383_MOESM1_ESM.docx]

Supplementary 1. Mosquito collection information in 90 sampling sites, Foshan, China, 2025

| Sampling site No. | longitude and latitude | Town | Environmental type | Sampling date | Mosquito collection | | |  |
| --- | --- | --- | --- | --- | --- | --- | --- | --- |
|  |  |  |  |  | No. of mosquito | No. of *Ae. albopictus* | No. of Female*Ae. albopictus* | |
| S1 | 22.955475,113.241928,-1.79 | Chencun | Parklands | 2025.07.31 | 30 | 26(0.87) | 17(0.65) | |
| S2 | 22.953634,113.229721,1.17 | Chencun | Parklands | 2025.07.31 | 0 | 0(0) | 0(0) | |
| S3 | 22.949833,113.230072,-2.82 | Chencun | Residences | 2025.07.31 | 36 | 34(0.94) | 15(0.42) | |
| S4 | 22.947151,113.233315,-2.22 | Chencun | Residences | 2025.07.31 | 6 | 5(0.83) | 5(0.83) | |
| S5 | 22.945978,113.238266,1.36 | Chencun | Parklands | 2025.07.31 | 51 | 51(1) | 44(0.86) | |
| S6 | 22.957052,113.238411,-3.34 | Chencun | Parklands | 2025.07.31 | 85 | 84(0.99) | 19(0.22) | |
| S7 | 22.961157,113.236382,3.09 | Chencun | Parklands | 2025.07.31 | 9 | 7(0.78) | 5(0.56) | |
| S8 | 22.964167,113.227379,4.74 | Chencun | Parklands | 2025.07.31 | 37 | 30(0.81) | 5(0.14) | |
| S9 | 22.957512,113.227165,-1.71 | Chencun | Parklands | 2025.07.31 | 21 | 10(0.48) | 5(0.24) | |
| S10 | 22.959925,113.221344,-4.42 | Chencun | Residences | 2025.07.31 | 48 | 48(1) | 40(0.83) | |
| S11 | 22.972282,113.112000,6.72 | Lecong | Parklands | 2025.08.01 | 160 | 160(1) | 59(0.37) | |
| S12 | 22.972204,113.107788,2.21 | Lecong | Parklands | 2025.08.01 | 9 | 9(1) | 8(0.89) | |
| S13 | 22.973234,113.097481,-0.84 | Lecong | Residences | 2025.08.01 | 22 | 16(0.73) | 5(0.23) | |
| S14 | 22.973757,113.096970,-3.98 | Lecong | Residences | 2025.08.01 | 36 | 26(0.72) | 22(0.61) | |
| S15 | 22.976496,113.093040,-0.01 | Lecong | Residences | 2025.08.01 | 74 | 74(1) | 54(0.73) | |
| S16 | 22.975836,113.092888,-4.01 | Lecong | Residences | 2025.08.01 | 37 | 36(0.97) | 29(0.78) | |
| S17 | 22.965343,113.089172,-2.75 | Lecong | Parklands | 2025.08.01 | 23 | 11(0.48) | 6(0.26) | |
| S18 | 22.959852,113.100372,0.09 | Lecong | Parklands | 2025.08.01 | 45 | 42(0.93) | 32(0.71) | |
| S19 | 22.963625,113.104378,-8.28 | Lecong | Parklands | 2025.08.01 | 13 | 10(0.77) | 6(0.46) | |
| S20 | 22.956902,113.093819,1.90 | Lecong | Parklands | 2025.08.01 | 10 | 10(1) | 8(0.8) | |
| S21 | 22.965977,113.085014,-1.06 | Lecong | Parklands | 2025.08.02 | 9 | 9(1) | 7(0.78) | |
| S22 | 22.957771,113.080109,-1.66 | Lecong | Parklands | 2025.08.02 | 4 | 4(1) | 1(0.25) | |
| S23 | 22.957047,113.077057,0.69 | Lecong | Parklands | 2025.08.02 | 14 | 14(1) | 9(0.64) | |
| S24 | 22.946693,113.075287,-2.69 | Lecong | Residences | 2025.08.02 | 1 | 1(1) | 1(1) | |
| S25 | 22.938643,113.079262,-1.06 | Lecong | Parklands | 2025.08.02 | 50 | 48(0.96) | 27(0.54) | |
| S26 | 22.923597,113.070625,-1.71 | Lecong | Parklands | 2025.08.02 | 34 | 34(1) | 8(0.24) | |
| S27 | 22.911486,113.090263,-3.43 | Lecong | Parklands | 2025.08.02 | 34 | 34(1) | 15(0.44) | |
| S28 | 22.939260,113.093658,-4.03 | Lecong | Residences | 2025.08.02 | 1 | 1(1) | 1(1) | |
| S29 | 22.941837,113.095718,- 10.78 | Lecong | Parklands | 2025.08.02 | 86 | 55(0.64) | 19(0.22) | |
| S30 | 22.950142,113.094528,-11.59 | Lecong | Residences | 2025.08.02 | 22 | 22(1) | 12(0.55) | |
| S31 | 22.963081,113.116425,0.54 | Lecong | Parklands | 2025.08.03 | 27 | 20(0.74) | 14(0.52) | |
| S32 | 22.955696,113.115623,-5.01 | Lecong | Residences | 2025.08.03 | 6 | 6(1) | 5(0.83) | |
| S33 | 22.955128,113.117287,-4.74 | Lecong | Parklands | 2025.08.03 | 31 | 31(1) | 19(0.61) | |
| S34 | 22.945007,113.105316,-4.06 | Lecong | Residences | 2025.08.03 | 6 | 6(1) | 2(0.33) | |
| S35 | 22.940918,113.105118,-4.17 | Lecong | Parklands | 2025.08.03 | 22 | 20(0.91) | 13(0.59) | |
| S36 | 22.940413,113.099709,-2.79 | Lecong | Residences | 2025.08.03 | 45 | 45(1) | 24(0.53) | |
| S37 | 22.936750,113.110977,-5.83 | Lecong | Residences | 2025.08.03 | 16 | 16(1) | 12(0.75) | |
| S38 | 22.932940,113.113350,-5.53 | Lecong | Parklands | 2025.08.03 | 14 | 13(0.93) | 11(0.79) | |
| S39 | 22.932940,113.113350,-5.53 | Lecong | Parklands | 2025.08.03 | 17 | 17(1) | 13(0.76) | |
| S40 | 22.932940,113.113350,-5.53 | Lecong | Parklands | 2025.08.03 | 22 | 22(1) | 11(0.5) | |
| S41 | 22.963381,113.143616,3.23 | Lecong | Parklands | 2025.08.04 | 19 | 19(1) | 18(0.95) | |
| S42 | 22.968616,113.131584,4.26 | Lecong | Parklands | 2025.08.04 | 54 | 54(1) | 40(0.74) | |
| S43 | 22.969074,113.119949,-0.59 | Lecong | Parklands | 2025.08.04 | 41 | 30(0.73) | 21(0.51) | |
| S44 | 22.963629,113.131615,-7.78 | Lecong | Residences | 2025.08.04 | 8 | 8(1) | 5(0.63) | |
| S45 | 22.965038,113.127007,-5.94 | Lecong | Parklands | 2025.08.04 | 47 | 47(1) | 28(0.6) | |
| S46 | 22.959116,113.124023，-1.38 | Lecong | Residences | 2025.08.04 | 19 | 14(0.74) | 12(0.63) | |
| S47 | 22.959806,113.119278,0.45 | Lecong | Residences | 2025.08.04 | 26 | 25(0.96) | 14(0.54) | |
| S48 | 22.956518,113.128998,-1.48 | Lecong | Parklands | 2025.08.04 | 8 | 8(1) | 7(0.88) | |
| S49 | 22.948227,113.131241,2.10 | Lecong | Parklands | 2025.08.04 | 14 | 9(0.64) | 9(0.64) | |
| S50 | 22.938456,113.122124,-0.55 | Lecong | Parklands | 2025.08.04 | 23 | 18(0.78) | 11(0.48) | |
| S51 | 22.952625,113.143990，-0.38 | Lecong | Parklands | 2025.08.05 | 28 | 28(1) | 18(0.64) | |
| S52 | 22.949583,113.137161,-2.05 | Lecong | Residences | 2025.08.05 | 20 | 15(0.75) | 13(0.65) | |
| S53 | 22.956423,113.132774,-1.59 | Lecong | Residences | 2025.08.05 | 37 | 36(0.97) | 21(0.57) | |
| S54 | 22.934568,113.134209,-2.14 | Lecong | Parklands | 2025.08.05 | 1 | 1(1) | 1(1) | |
| S55 | 22.933887,113.137299,-2.47 | Lecong | Parklands | 2025.08.05 | 11 | 9(0.82) | 8(0.73) | |
| S56 | 22.931562,113.134583,-6.87 | Lecong | Parklands | 2025.08.05 | 12 | 12(1) | 10(0.83) | |
| S57 | 22.929783,113.129494,-2.56 | Lecong | Parklands | 2025.08.05 | 8 | 8(1) | 7(0.88) | |
| S58 | 22.927799,113.131149,3.75 | Lecong | Parklands | 2025.08.05 | 9 | 6(0.67) | 5(0.56) | |
| S59 | 22.923870,113.141296,1.57 | Lecong | Parklands | 2025.08.05 | 5 | 4(0.8) | 3(0.6) | |
| S60 | 22.927221,113.141434,0.54 | Lecong | Residences | 2025.08.05 | 12 | 12(1) | 3(0.25) | |
| S61 | 22.958267,113.146805,3.02 | Beijiao | Parklands | 2024.08.06 | 35 | 33(0.94) | 13(0.37) | |
| S62 | 22.952589,113.154701,-0.96 | Beijiao | Parklands | 2024.08.06 | 11 | 10(0.91) | 6(0.55) | |
| S63 | 22.943665,113.147926,3.20 | Beijiao | Parklands | 2024.08.06 | 8 | 6(0.75) | 4(0.5) | |
| S64 | 22.942019,113.145805,-7.25 | Beijiao | Parklands | 2024.08.06 | 31 | 30(0.97) | 9(0.29) | |
| S65 | 22.934780,113.165100,-5.23 | Beijiao | Residences | 2024.08.06 | 16 | 16(1) | 6(0.38) | |
| S66 | 22.931213,113.165192,4.31 | Beijiao | Residences | 2024.08.06 | 7 | 7(1) | 3(0.43) | |
| S67 | 22.924730,113.158623,-1.30 | Beijiao | Parklands | 2024.08.06 | 37 | 34(0.92) | 14(0.38) | |
| S68 | 22.917967,113.147591,2.80 | Beijiao | Parklands | 2024.08.06 | 47 | 46(0.98) | 24(0.51) | |
| S69 | 22.921291,113.152603,1.91 | Beijiao | Residences | 2024.08.06 | 9 | 9(1) | 4(0.44) | |
| S70 | 22.912149,113.161278,1.77 | Beijiao | Parklands | 2024.08.06 | 24 | 24(1) | 19(0.79) | |
| S71 | 22.951502,113.176147,4.86 | Beijiao | Parklands | 2025.08.09 | 28 | 28(1) | 20(0.71) | |
| S72 | 22.929945,113.196190,-5.67 | Beijiao | Parklands | 2025.08.09 | 141 | 138(0.98) | 82(0.58) | |
| S73 | 22.919039,113.199074,-1.49 | Beijiao | Parklands | 2025.08.09 | 56 | 56(1) | 42(0.75) | |
| S74 | 22.919886,113.204460,-6.68 | Beijiao | Parklands | 2025.08.09 | 18 | 17(0.94) | 16(0.89) | |
| S75 | 22.903912,113.209663,-0.82 | Beijiao | Residences | 2025.08.09 | 79 | 78(0.99) | 57(0.72) | |
| S76 | 22.901756,113.198616,-2.17 | Beijiao | Parklands | 2025.08.09 | 15 | 15(1) | 8(0.53) | |
| S77 | 22.901625,113.180038,0.27 | Beijiao | Parklands | 2025.08.09 | 13 | 12(0.92) | 10(0.77) | |
| S78 | 22.909956,113.180351,-7.29 | Beijiao | Residences | 2025.08.09 | 125 | 117(0.94) | 78(0.62) | |
| S79 | 22.926691,113.174591,-17.54 | Beijiao | Parklands | 2025.08.09 | 28 | 25(0.89) | 23(0.82) | |
| S80 | 22.938677,113.185883,-3.38 | Beijiao | Parklands | 2025.08.09 | 57 | 57(1) | 27(0.47) | |
| S81 | 22.955221,113.184517，-2.29 | Beijiao | Parklands | 2025.08.10 | 30 | 28(0.93) | 20(0.67) | |
| S82 | 22.947800,113.204201,-9.52 | Beijiao | Parklands | 2025.08.10 | 61 | 61(1) | 33(0.54) | |
| S83 | 22.940449,113.226410,-9.86 | Beijiao | Parklands | 2025.08.10 | 72 | 69(0.96) | 44(0.61) | |
| S84 | 22.934334,113.207855,-8.54 | Beijiao | Parklands | 2025.08.10 | 27 | 24(0.89) | 13(0.48) | |
| S85 | 22.930031,113.224838,-11.53 | Beijiao | Parklands | 2025.08.10 | 6 | 3(0.5) | 1(0.17) | |
| S86 | 22.937695,113.239998,5.73 | Beijiao | Parklands | 2025.08.10 | 50 | 47(0.94) | 23(0.46) | |
| S87 | 22.969385,113.187569,-0.87 | Chencun | Parklands | 2025.08.10 | 88 | 81(0.92) | 60(0.68) | |
| S88 | 22.982071,113.156708,-9.29 | Chencun | Parklands | 2025.08.10 | 38 | 37(0.97) | 20(0.53) | |
| S89 | 22.972738,113.155708,-10.71 | Chencun | Parklands | 2025.08.10 | 4 | 3(0.75) | 3(0.75) | |
| S90 | 22.975294,113.138359,-12.01 | Chencun | Parklands | 2025.08.10 | 47 | 46(0.98) | 25(0.53) | |

|  |  |  |  |
| --- | --- | --- | --- |
